# Opiophobia in Emergency Department Healthcare Providers: A Survey in Western Switzerland

**DOI:** 10.3390/jcm10071353

**Published:** 2021-03-25

**Authors:** Sarah Bertrand, Gabriel Meynet, Patrick Taffé, Vincent Della Santa, Daniel Fishman, Yvan Fournier, Vincent Frochaux, Vincent Ribordy, Olivier T. Rutschmann, Olivier Hugli

**Affiliations:** 1 Faculty of Biology and Medicine, University of Lausanne, 1011 Lausanne, Switzerland; sarah.bertrand@chuv.ch (S.B.); gabriel.meynet@chuv.ch (G.M.); 2 Institute of Social and Preventive Medicine, University of Lausanne, Corniche 10, 1010 Lausanne, Switzerland; Patrick.Taffe@chuv.ch; 3 Emergency Department, Hôpital Neuchâtelois, Maladière 45, 2000 Neuchâtel, Switzerland; Vincent.DellaSanta@h-ne.ch; 4 Emergency Department, Route de Morgins, Hôpital du Chablais, 1870 Monthey, Switzerland; daniel.fishman@soignez-moi.ch; 5 Emergency Department, Colline 3, Hôpital Intercantonal de la Broye, 1530 Payerne, Switzerland; yvan.fournier@hibroye.ch; 6 Emergency Department, Grand-Champsec 80, Hôpital de Sion, 1950 Sion, Switzerland; vincent.frochaux@hopitalvs.ch; 7 Emergency Department, Pensionnats 2-6, Hôpital Fribourgeois, 1708 Fribourg, Switzerland; vincent.ribordy@h-fr.ch; 8 Emergency Department, Geneva University Hospitals and School of Medicine, Gabrielle Perret-Gentil 2, 1205 Geneva, Switzerland; orutschmann@hotmail.com; 9 Emergency Department, University Hospital of Lausanne and Lausanne University, Bugnon 46, 1011 Lausanne, Switzerland

**Keywords:** acute pain, opiates, opiophobia, morphine, emergency department, uncertainty, risk-taking, risks

## Abstract

Opiophobia contributes to oligoanalgesia in the emergency department (ED), but its definition varies, and its association to healthcare providers’ personality traits has been scantly explored. Our purpose was to study the different definitions of opiophobia and their association with two personality traits of doctors and nurses working in EDs, namely the stress from uncertainty and risk-taking. We used three online questionnaires: the ‘Attitude Towards Morphine Use’ Score (ATMS), the Stress From Uncertainty Scale (SUS) and the Risk-Taking Scale (RTS). Doctors and nurses from nine hospital EDs in francophone Switzerland were invited to participate. The ATMS score was analyzed according to demographic characteristics, SUS, and RTS. The response rate was 56%, with 57% of respondents being nurses and 63% women. Doctors, less experienced and non-indigenous participants had a significantly higher ATMS (all *p* ≤ 0.01). The main contributors of the ATMS were the fear of side effects and of addiction. In multivariate analysis, being a doctor, less experience and non-indigenous status were predictive of the ATMS; each point of the SUS increased the ATMS by 0.24 point. The fear of side effects and of addiction were the major contributors of opiophobia among ED healthcare providers; opiophobia was also associated with their personality traits.

## 1. Introduction

Pain relief is one of the priority tasks of the emergency department (ED). However, inadequate analgesia, or oligoanalgesia, remains prevalent in this setting, especially for patients with severe acute pain initially [1,2], and for which intravenous opiates are one of the treatment of choice [3]. However, opiates are sometimes simply not administered or only in subtherapeutic doses [4]. This reluctance to use narcotic analgesics has been reported in a majority of ED doctors [5], due to a lack of knowledge and training of ED doctors, or even prejudice against the use of opioid analgesics. This prejudice, or opiophobia, is defined variously in the literature as underutilization linked to the irrational fear of addiction [6], to an exaggerated fear of side effects [7], to moral reasons or to the legal risks associated with their prescription [8,9,10]. Whatever its cause, opiophobia contributes to oligoanalgesia [6,7].

Variation between doctors in pain management decisions is another cause of oligoanalgesia [2]. Wide variations in the rate of opioid administration between ED doctors have been demonstrated during ED stay, by clinical vignettes [11] or by chart review [12], or at the time of ED discharge [13]. Doctors’ personality is a significant determinant in practice variations. Individual tolerance to clinical uncertainty and to risks are factors influencing ED doctors’ decision-making [14,15,16]. Both these traits may be associated with opiophobia, which reflects an excessive risk perception associated with opioid use, in particular to treat a condition as uncertain as pain [17].

However, the relationship between stress related to uncertainty or risk and opiophobia has not yet been investigated among healthcare providers in the ED. The primary goal of our study was therefore to characterize and quantify the various components of opiophobia amongst doctors and nurses working in the main hospital-based EDs in francophone Switzerland, and study their association with doctors’ and nurses’ demographic and professional characteristics, as well as their tolerance to uncertainty and risk. Regarding the latter two, our assumption was that there would be a positive correlation between the degree of opiophobia and intolerance to uncertainty, whereas this correlation would be negative with risk tolerance.

## 2. Materials and Methods

The study was exempted from formal review by the Human Research Ethics Committee of the State of Vaud based on the Swiss Federal Act on Research involving Human Beings, because the survey was anonymous, voluntary, and without health-related data.

### 2.1. Survey Recruitment and Instrument

Data were collected from a web-based survey between September 2016 and April 2017 from ED nurses and doctors of nine teaching hospitals of French-speaking Switzerland, two of which were university hospitals. The median number of patient visits in these EDs was 17300/year (range: 12,612–60,500). Survey questions included demographics and three validated questionnaires: (1) the “Attitudes Towards Morphine” Score (ATMS) [18], composed of 19 statements related to the use of morphine and grouped into five subscales: risk of addiction/dependence, operational reasons for not using morphine, risk of escalating doses, risks other than addiction and other non-operational reasons (Appendix A); (2), the translation, validated in French, of the ‘Stress from Uncertainty Scale’ (SUS), which evaluates the emotional reaction in the face of uncertainty [15,19], and is composed of eight items grouped into two subscales: the anxiety due to uncertainty and the concern about bad outcome; (3) the Risk Taking Scale (RTS), a six-item scale adapted from the Jackson Personality Index evaluating general risk-taking behavior [16]. All items were rated on a 5-point Likert scale anchored by 1 (totally disagree) and 5 (totally agree), with a possible score distribution between 19 and 95 points for the ATMS, 8 to 40 points for SUS and 6 to 30 for RTS. Scores of negatively worded items in the SUS or indicating risk averseness in the RTS were reversed so that a higher score represented a greater stress from uncertainty and a greater propensity to risk taking, respectively. We followed the Checklist for Reporting Results of Internet E-Surveys (CHERRIES) to report our methodology (Appendix B).

### 2.2. Data Collection

The questionnaire was available online on the platform SurveyMonkey^®^ (SurveyMonkey Inc, San Mateo, CA, USA). Head doctors of each ED emailed to every nurse or doctor an information letter inviting them to participate in the study, containing the internet link of the questionnaire and a unique personal identifier. After accessing their personal web-link, participants first underwent an introductory screen providing information about the study, and asking for their explicit consent to participate. Consent was indicated when respondents clicked on the ‘Go to Survey’ button from this page. A reminder was sent after 2 and 4 months to non-responders. There were no exclusion criteria to the study, and no incentive was offered.

### 2.3. Statistical Analysis

The descriptive analyses are presented as average and standard deviations (SD), median and interquartile range (IQR) or percentages. Comparisons between groups were performed by Student t-test, analysis of variance (ANOVA) or Wilcoxon rank-sum test, as appropriate. The correlation between continuous variables was determined by Pearson coefficient. The primary outcome was the ATMS, and the secondary outcomes were its five subscales. Multivariable linear regression analysis was used to assess the association between the various outcomes and the following regressors: gender, nurse/doctor, number of years of experience (in quintiles), number of years of experience in an emergency service (in quintiles), hospital (categorical variable), nationality (Swiss, European, extra-European, missing), pain training (yes/no), the RTS, and SUS. A backward elimination procedure was used to select the final model. The categorical variable Hospital was used as a fixed effect to account for the clustering of data within hospitals. Interactions between the dichotomous variable nurse/doctor and number of years of experience, SUS, and pain training, were assessed by Wald test and goodness of fit by residual analysis. The best functional form for the two RTS and SUS scores was determined using the method of fractional polynomials outcomes [20]. To pinpoint the contribution of the various regressors to the primary outcome, the same set of regressors as selected for the primary outcome was assessed for the secondary outcomes. The coefficients represent the change in points of the score associated with each variable. All questionnaires, including those terminated early, were analyzed, and missing data were not imputed. All data analyses were carried out using Stata, v14 (StataCorp, College Station, TX, USA) and a bilateral *p* value < 0.05 considered significant.

## 3. Results

The invitation to fill the questionnaire was sent to 916 healthcare providers (372 doctors and 544 nurses). In total, 511 surveys were completed online, with a response rate of 56%, and with significant variation between centers: from 28% to 85% (*p* < 0.001). The rate was similar between doctors (57%) and nurses (52%) (*p* = 0.16); 16 respondents (3%) did not indicate their profession. The majority of the questionnaires came from nurses (Table 1).

Two-thirds of the participants were women, with a greater proportion among nurses (*p* < 0.001). Nurses also had greater total as well as ED postgraduate experience (*p* < 0.001). Most doctors, but less than one out of ten nurses, reported having received any training on pain management (*p* < 0.001). Half of healthcare providers, and a large majority of doctors, were Swiss, while a majority of nurses were European (*p* < 0.001). France was the single most frequent country of origin, with 41% of nurses and 12% of doctors. Nearly a third of nurses and half of doctors worked in a university ED (*p* < 0.001).

The ATMS was 44.7 ± 8.7 points (range 24–69) (Table 1). ATMS and SUS were significantly higher among doctors than nurses; there was no difference for the RTS. There was a weak negative but significant correlation between the SUS and RTS (r = −0.18; *p* = 0.0001), and between years of experience and the SUS (r = −0.26; *p* > 0.001), but not with the RTS. A positive correlation existed between the ATMS and SUS (r = 0.30; *p* < 0.001), found among doctors (r = 0.33; *p* < 0.001) as well as nurses (r = 0.24; *p* < 0.001). However, no association was found between the ATMS and the RTS.

The ATMS and its five subscales were associated with the healthcare providers’ characteristics (Table 2).

ATMS was 6.2 and 4.7 points higher among those with ≤10 years of total and ≤4 years of ED postgraduate experience, respectively. ATMS was also higher among healthcare providers of European origin or outside of Europe. On the other hand, gender, and ED site or the academic status of the hospital were not associated with the ATMS.

**Figure 1:**
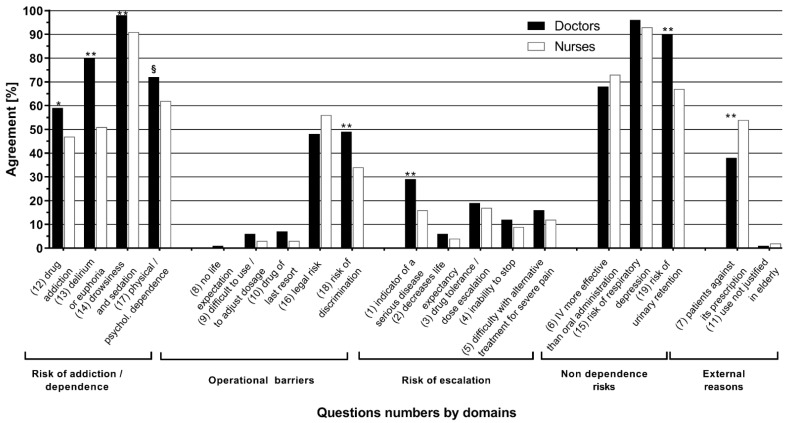
Percentage of agreement for each dichotomized item of the “Attitude Towards Morphine Score” grouped by profession and subscale. Agreement corresponds to the values of 1 and 2 of the Likert scale (i.e., Totally Agree or Agree); *: *p* <0.01; **: *p* ≤ 0.001; §: *p* < 0.05 for comparisons between MDs et RNs. Numbers in brackets refer to the question numbers in the Appendix A.

At the level of the five ATMS subscales (Table 2), doctors scored significantly higher than nurses on each of the subscales, except the operational one, and differences were found based on total postgraduate and ED experience, except for the external risks. On the other hand, there were only differences in the operational and the external risk subscales based on origin. In dichotomizing the responses (Figure 1), it is apparent that the subscales concerning risk perception, related to addiction or not, consistently reached a majority and contributed significantly to the increase in the ATMS. Doctors were more often in agreement than nurses with questions related to risk of addiction (all *p* < 0.05).

The changes in points of the ATMS and its subscale scores associated with the variables included in our final model are presented in Table 3. Profession was predictive of the total ATMS (minus 2.4 points for nurses), as well as the risk of addiction, other risks, and non-operational subscales. The greater the number of years of postgraduate training, the lower the ATMS (−6 points for the most experiences group), or its subscales scores were, except for the ‘other risks’ subscale. An indigenous status was also a predictor of a lower ATMS, but less systematically so for subscales. Each additional point of the SUS increased the ATMS by a quarter of a point, and so contributed from 6 to 17 points in our model. Our whole model explained 24% of the total variation in the score (*p* < 0.001).

## 4. Discussion

Opiophobia is one of the causes of the underutilization of opiates to treat severe pain in the ED, and a contributor to oligoanalgesia. In our study, the administration of morphine was associated with fears of addiction and dangerous side effects. These fears were more pronounced among doctors and, in all caregivers, related to a shorter work experience. Addiction and the opioid crisis affecting the USA have been widely relayed in the medical press in recent years [21]. Although this crisis is essentially caused by the overprescription of opioids for chronic pain, the increasing prescription of an opioid at the time of ED discharge over the last two decades is now recognized as a risk of future addiction [22]. However, the risk of addiction following the administration of opioids during the ED visit only is less clear. Nevertheless, awareness of this risk is important for ED doctors and should lead to responsible prescription, but not at the expense of a greater prevalence of oligoanalgesia [23,24].

Fears of sedation and respiratory depression are also widespread, both among healthcare providers and the general population [9,10,18,25,26]. Opioid-induced respiratory depression is rare but can be fatal. The administration of the smallest effective dose to relieve pain is therefore essential to minimize this risk. Recommended opioid regimen, given either as a bolus or even better, titrated to determine effective pain relief, have little risk of respiratory depression [27]. Better teaching of opioid pharmacology could correct this exaggerated risk perception. The impact of lectures is, however, limited, since the prescription of opioids is primarily by customary learning, where healthcare providers learn and adopt the teaching at the bedside provided by their cohesive peer group [6]. The association of opiophobia with healthcare providers’ indigenous status and with their professional experience may be viewed as a reflection of such customary learning behavior. Attitudes towards morphine differ between countries; the majority of our non-indigenous healthcare providers were from neighboring France, where opioids are less commonly used than in Switzerland [26,28]. As our data shows, it takes years to diminish ingrained fears, and thus there is a need for different educational interventions. Our study shows an association between the SUS and the ATMS. Even though this association does not demonstrate a causal link nor is proof of a lower opioid prescription rate, we showed recently that nurses with a higher SUS were less likely to use a nurse-led pain protocol [29]. SUS may prove to be a new malleable target for educational interventions [30], and an innovative approach to reduce the burden of unrelieved pain. However, the magnitude of change in pain management induced by such an intervention needs to the focus of additional research.

While the RTS was associated with referral decisions and biological or radiological testing in other studies [14,16], no correlation between RTS and opiophobia was found. While these previous studies addressed the association between tests or decisions performed, ours studied the link between a propensity to risk and concern of using morphine. We cannot exclude the fact that a correlation may exist between RTS and actual opioid prescription patterns.

Our study has several limitations. First, it did not investigate the actual use of morphine, and there may be significant differences between the expressed degree of opiophobia and the administration of opioids in real practice. Secondly, the study was limited to francophone Switzerland and cannot necessarily be extrapolated to EDs elsewhere in Switzerland or abroad. Thirdly, although our response rate was 56%, and higher than that often obtained in this type of survey, non-participants might differ from respondents. It was not possible to know their characteristics, as the survey was anonymous. Finally, the authors of the scale have not provided a specific cut-off, above which one becomes “opiophobic”. However, the higher the score, the greater the concern is to use opioids. In addition, the minimal clinically relevant difference of the score, i.e., representative of a true change in attitude and not just a statistically significant difference, is not defined. Compared to the scores in the two studies published previously [9,18], the total score in our study was similar for physicians, but four points lower for nurses. If these differences correspond to actual differences in pain management at the bedside remains an open question.

## 5. Conclusions

Opiophobia of health care providers working in EDs of francophone Switzerland expressed mainly the fear of side-effects and addiction. It was associated with individual stress related to clinical uncertainty but not with risk tolerance. If a causal link between the SUS and the prescription of opioids were demonstrated, a better ability to manage uncertainty could lead to better use of opioids in the ED to treat acute pain.

## Figures and Tables

**Table 1:** Sociodemographic characteristics by professional category.

	All * (N = 511)	Nurses (N = 283)	Doctors (N = 212)	*p*-Value
Women, *n* (%)	311 (63)	203 (72)	108 (51)	<0.001
Median postgraduate experience:				
Total, years (IQR) ^π^	10 (4; 17)	12 (7; 20)	5.5 (2; 12)	<0.001
In the ED, years (IQR)	4 (1; 10)	7 (3; 14)	2 (1; 5)	<0.001
Pain management training, *n* (%)	104 (21)	20 (7.1)	84 (81)	<0.001
Nationality				<0.001
Swiss, *n* (%)	260 (51)	123 (43)	137 (65)	
European, *n* (%)	217 (42)	152 (54)	65 (31)	
Non European, *n* (%)	14 (2.9)	7 (2.5)	8 (3.8)	
Missing, *n* (%)	19 (3.7)	1 (0.4)	2 (0.9)	
Hospital,				<0.001
University, *n* (%)	167 (32)	74 (26)	88 (42)	
Non university, *n* (%)	271 (53)	169 (60)	94 (44)	
unknown, *n* (%)	73 (14)	40 (14)	30 (14)	
Mean scores				
ATMS ^¶^, points (SD **)	44.7 (8.7)	43.0 (8.5)	46.5 (8.5)	<0.001
SUS ^§^, points (SD)	22.6 (6.7)	21.6 (6.4)	23.9 (6.9)	<0.001
RTS ^¥^, points (SD)	14.1 (4.1)	14.0 (4.1)	14.1 (4.0)	0.55

Due to rounding, totals may not always add up to 100%. *: the sum is greater than the total of nurses and doctors, as 16 participants did not indicate their profession. ^π^: Interquartile range. ^¶^: ATMS = Attitude Towards Morphine Score. ^§^: SUS = Stress from Uncertainty Score. ^¥^: RTS = Risk Taking Scale. **: Standard deviation.

**Table 2:** Association between the Attitude towards Morphine Score (ATMS), its 5 subscales, and the healthcare providers’ characteristics.

	ATMS, Points (SD)	Subscales Points (SD)				
		Addiction/ Dependence	Operational	Escalation	Non-Dependence Risks	External Risks
Profession						
Nurse	43.0 (8.5)	12.8 (3.8)	8.0 (2.4)	7.4 (2.9)	11.1 (1.9)	3.8 (1.2)
Doctor	46.9 (8.5)	14.8 (3.6)	8.4 (2.6)	8.4 (3.1)	11.9 (2.3)	3.5 (1.1)
*p*-value	<0.001	<0.001	0.06	0.005	<0.001	<0.001
Postgraduate experience *						
0–3 years	49.4 (8.1)	15.2 (3.3)	9.2 (2.7)	9.5 (3.4)	11.7 (2.1)	3.9 (1.2)
4–7 years	46.1 (8.2)	14.0 (3.8)	8.2 (2.2)	8.3 (2.8)	11.9 (1.9)	3.7 (1.1)
8–12 years	44.2 (7.4)	13.8 (3.4)	7.8 (2.3)	7.5 (3.0)	11.5 (2.0)	3.6 (1.0)
13–19 years	41.7 (9.1)	12.8 (4.2)	7.8 (2.5)	6.7 (2.3)	11.0 (2.5)	3.5 (1.1)
20–39 years	41.2 (8.0)	12.1 (3.7)	7.5 (2.5)	6.9 (2.6)	10.8 (2.4)	3.8 (1.3)
*p*-value	<0.001	<0.001	<0.001	<0.001	0.001	0.099
ED experience *						
0–4 years	46.9 (8.4)	14.5 (3.5)	8.5 (2.5)	8.5 (3.2)	11.7 (2.0)	3.7 (1.1)
>4 years	42.2 (8.3)	12.7 (3.9)	7.7 (2.4)	7.1 (2.7)	11.1 (2.4)	3.6 (1.1)
*p*-value	<0.001	<0.001	<0.001	<0.001	0.006	0.37
Origin						
Switzerland	43.7 (8.6)	13.4 (3.8)	7.9 (2.3)	7.6 (2.9)	11.2 (2.2)	3.6 (1.1)
Europe	45.5 (8.6)	13.8 (3.8)	8.3 (2.6)	8.0 (3.0)	11.6 (2.2)	3.8 (1.2)
Extra-European	49.3 (9.9)	14.7 (4.7)	9.7 (3.6)	8.9 (3.8)	11.8 (2.8)	4.1 (1.1)
Unknown	47.3 (10.4)	14.8 (2.9)	8.8 (3.0)	7.0 (3.7)	11.8 (1.7)	4.3 (1.7)
*p*-value	0.01	0.29	0.02	0.13	0.14	0.04

*: Postgraduate professional experience dichotomized based on the median value.

**Table 3:** Multivariate model predicting the “Attitude Towards Morphine Score (ATMS) and the scores of its 5 subscales.

Variables	Total		Addiction Risk		Operational		Escalation		Other Risks		Non Operational	
	* Coefficients (95% CI)	*p*	* Coefficients (95% CI)	*p*	* Coefficients (95% CI)	*p*	* Coefficients (95% CI)	*p*	* Coefficients (95% CI)	*p*	* Coefficients (95% CI)	*p*
Intercept	44.3 (40.1–48.1)	<0.001	15.4 (13.7–17.2)	<0.001	7.5 (6.3–8.7)	<0.001	8.1 (6.7–9.4)	<0.001	10.4 (9.3–11.4)	<0.001	2.9 (2.4–3.5)	<0.001
Profession												
Doctor	Réf.	0.004	Réf.	<0.001	Réf.	0.83	Réf.	0.15	Réf.	0.01	Réf.	<0.001
Nurse	−2.4 (−4.0–−0.7)		−1.7 (−2.5–−1.0)		0.5 (−0.4–0.5)		−0.4 (−1.0–0.2)		−0.8 (−1.2–−0.3)		0.5 (0.2–0.7)	
Postgraduate experience												
0–3 years	Réf.		Réf.		Réf.		Réf.		Réf.		Réf.	
4–7 years	−2.5 (− 4.8–−0.3)	0.03	−0.8 (−1.8–0.25)	0.14	−0.9 (−1.6–−0.2)	0.01	−0.9 (−1.7–−0.1)	0.03	0.3 (0.2–0.9)	0.27	−0.3 (−0.6–0.02)	0.07
8–12 years	−4.3 (−6.6–−2.0)	<0.001	−1.1 (−2.1–0.1)	0.06	−1.3 (−2.0–−0.6)	<0.001	−1.7 (−2.5–−0.8)	<0.001	0.1 (−0.6–0.7)	0.82	−0.4 (−0.7–−0.1)	0.02
13–19 years	−6.1 (−8.5–−3.7)	<0.001	−1.8 (−2.9–−0.7)	0.001	−1.1 (−1.8–−0.3)	0.005	−2.4 (−3.3–−1.6)	<0.001	−0.3 (−1.0–0.3)	0.31	−0.4 (−0.8–−0.1)	0.01
20–39 years	−6.0 (−8.4–−3.6)	<0.001	−2.1 (−3.2–−1.0)	<0.001	−1.4 (−2.1–−0.6)	<0.001	−2.0 (−2.9–−1.1)	<0.001	−0.4 (−1.1–0.2)	0.21	−0.2 (−0.5–0.2)	0.36
Nationality												
Swiss	Réf.		Réf.		Réf.		Réf.		Réf.		Réf.	
European	2.5 (1.0–4.0)	0.001	0.8 (0.1–3.0)	0.03	0.3 (−0.1–0.8)	0.15	0.7 (0.2–1.3)	0.01	0.6 (0.2–1.0)	0.006	0.1 (−0.1–0.3)	0.49
Non-European	4.7 (0.6–8.8)	0.02	1.1 (−0.8–3.0)	0.27	1.7 (0.5–3.0)	0.007	1.1 (−0.3–2.6)	0.13	0.3 (−0.8–1.5)	0.55	0.5 (−0.1–1.0)	0.12
Unknown	17.5 (1.6–33.3)	0.03	3.3 (−4.0–10.7)	0.38	0.1 (−4.7–5.0)	0.96	9.5 (3.9–15.1)	0.001	1.7 (−2.6–6.0)	0.44	2.9 (0.7–5.1)	0.01
SUS ^¶^ (for each additional point)	0.24 (0.13–0.35)	<0.001	0.03 (−0.02–0.08)	0.25	0.1 (0.04–0.11)	<0.001	0.06 (0.02–0.10)	0.002	0.04 (0.01–0.07)	0.013	0.04 (0.02–0.5)	<0.001

Model also adjusted for the ED center. *: Coefficients represents the beta coefficients from the linear regression analysis; ^¶^ SUS: Stress from uncertainty score.

## Data Availability

The data presented in this study are available on request from the corresponding author. The data are not publicly available, as participants of this study did not agree for their data to be shared publicly.

## Appendix A

**Table A1:** Question numbers included in each of the 5 subscales of the Attitude Towards Morphine Score (ATMS) and their total points.

Question N°	Components	
	Risk of addiction/dependence	20
12	Risk of drug addiction	
13	Risk of delirium or euphoria	
14	Risk of drowsiness and sedation	
17	Risk of physical and/or psychological dependence	
	Maximal points:	
	Operational reasons for not using morphine	25
8	The prescription of morphine means that there is no life expectation	
9	It is difficult to use and dose morphine	
10	Morphine is a drug of last resort	
16	Legal risk compared to other drugs	
18	Risk of discrimination	
	Maximal points:	
	Risk of escalation	
1	It means it is serious	
2	It decreases life expectancy	
3	The patient can get used to the drug quickly and one takes the risk of increasing the dose	
4	Once treatment is initiated, there is the risk of being unable to stop	
5	The early use of morphine makes it difficult to use any other treatment in severe pain	
	Maximal points:	25
	Other (nondependence) risks	15
6	IV administration is more effective than oral administration	
15	Risk of respiratory depression	
19	Risk of urinary retention	
	Maximal points:	
	External (nonoperational) reasons for not using morphine	10
7	The patients are against the prescription of morphine	
11	Sensation of pain decreases with age in the elderly, which does not justify its use	
	Maximal points:	
	Total points:	95

## Appendix B

**Table A2:** Checklist for Reporting Results of Internet E-Surveys (CHERRIES).

Checklist Item	Explanation		Line Number
Describe survey design	The target population was the emergency department (ED) nurses and doctors of nine teaching hospitals of French-speaking Switzerland and working in the ED at the time of the survey		81–83
Ethics	Ethics approval	The study was exempted from formal review on June 28th 2016 by a decision of the Human Research Ethics Committee of the State of Vaud based on the Swiss Federal Act on Research involving Human Beings, because the survey was anonymous, voluntary, and without health–related data.	77–79
	Informed consent	The welcome page presented briefly the goal of the study, that it would take approximately 15 min to complete, and that all responses were confidential and anonymous. Consent was indicated when respondents clicked on the ‘Go to Survey’ button from this page.	107–108
	Data protection	Data were collected using the online plateform SurveyMonkey^®^. No personal identifier was linked to survey results. The anonymous dataset was kept on password protected professional computers, behind an institutional firewall.	102–103
Development and testing	The survey instrument used three validated questionnaires. The survey was extensively tested by the authors to ensure that no typing errors existed.		84–98
Open survey versus closed survey	The survey was open and not password protected.		NA
Contact mode	Head doctors of each ED emailed to every nurse or doctor an information letter inviting them to participate in the study and containing the internet link of the questionnaire and a unique personal identifier		102–107
Advertising the survey	Head doctors of each ED emailed to every nurse or doctor an information letter inviting them to participate in the study and containing the internet link of the questionnaire and a unique personal identifier		102–107
Web/E-mail	This was a web-based survey, available online on the platform SurveyMonkey^®^		102
Context	The questionnaire was available online on the platform SurveyMonkey^®^		102
Mandatory/voluntary	it was a voluntary survey		102–108
Incentives	No incentives were offered		109
Time/Date	Data were collected between September 2016 and April 2017		81–82
Randomization of items or questionnaires	Items in the questionnaires were not randomized nor alternated		Not mentioned in manuscript
Adaptive questioning	Adaptive questioning was not used		Not mentioned in manuscript
Number of Items	Our survey presented 3 questionnaires: The ‘attitudes towards morphine’ score (ATMS) is composed of 19 statements related to the use of morphine and grouped into five subscales: risk of addiction/dependence, operational reasons for not using morphine, risk of escalating doses, risks other than addiction and other non-operational reasons. The translation, validated in French, of ‘Stress from uncertainty scale’ (SUS) which evaluates the emotional reaction in the face of uncertainty, composed of eight items grouped into two ‘subscales’: the anxiety due to uncertainty and the concern about bad outcome issues; The risk-taking scale (RTS), a six-item scale adapted from the Jackson Personality Index, which evaluates general risk-taking behavior A maximum of 12 items were displayed on any one survey page		84–93
Number of screens (pages)	The full survey was distributed over 6 screens		Not mentioned in manuscript
Completeness check	All survey items were mandatory. Completeness was checked after submission of each screen of the questionnaire had been submitted, and missing items were highlighted		Not mentioned in manuscript
Review step	Respondents were able to change their responses once submitted through a Back button		Not mentioned in manuscript
Unique site visitor	We did not determine unique visitors to ensure that respondents completed the survey only once		Not mentioned in manuscript
View rate (Ratio of unique survey visitors/unique site visitors)	Not applicable		
Participation rate (Ratio of unique visitors who agreed to participate/unique first survey page visitors)	Not applicable		
Completion rate	The invitation to fill the questionnaire was sent to 916 healthcare providers (372 doctors and 544 nurses). In total, 511 surveys were completed online, with a response rate of 56%, having significant variation between centers: from 28% to 85% (*p* < 0.001). The rate was similar between doctors (57%) and nurses (52%) (*p* = 0.16)		133–135
Cookies used	No		Not mentioned in manuscript
IP check	No		Not mentioned in manuscript
Log file analysis	No		Not mentioned in manuscript
Registration	No		Not mentioned in manuscript
Handling of incomplete questionnaires	All questionnaire, including those terminated early were analyzed		129–130
Questionnaires submitted with an atypical timestamp	No respondents were removed from the survey		Not mentioned in manuscript
Statistical correction	Not applied		Not mentioned in manuscript

This checklist has been modified from Eysenbach G. Improving the quality of Web surveys: the Checklist for Reporting Results of Internet E-Surveys (CHERRIES). J. Med. Internet Res. 29 September 2004, 6, e34 [erratum in J. Med. Internet Res. 2012, 14, e8.]. Article available at https://www.jmir.org/2004/3/e34/ (accessed on 24 March 2021); erratum available https://www.jmir.org/2012/1/e8/ (accessed on 24 March 2021). Copyright ©Gunther Eysenbach. Originally published in the Journal of Medical Internet Research, 29 September 2004 and 4 January 2012.
